# Complete Genome Sequence of *Roseibium* sp. Strain Sym1, a Bacterial Associate of Symbiodinium linucheae, the Microalgal Symbiont of the Anemone *Aiptasia*

**DOI:** 10.1128/mra.01118-22

**Published:** 2023-02-15

**Authors:** Emily G. Aguirre, Harold K. Carlson, Carly D. Kenkel

**Affiliations:** a Department of Biological Sciences, University of Southern California, Los Angeles, California, USA; Indiana University, Bloomington

## Abstract

We sequenced the genome of *Roseibium* sp. strain Sym1, a strain isolated from a monoculture of a Symbiodiniaceae marine dinoflagellate, Symbiodinium linucheae, a microalgal symbiont of cnidarians. The completed genome consists of one circular chromosome of 6,694,563 bp and four plasmids of 192,102 bp, 160,136 bp, 120,881 bp, and 89,413 bp.

## ANNOUNCEMENT

*Roseibium* species (formerly classified as *Labrenzia* [1, 2]) are part of the core microbiome of Symbiodiniaceae clades (3). Although several *Roseibium* genomes are available, it remains an understudied genus (1, 4, 5). We sequenced strain Sym1 to further explore the metabolic potential of *Roseibium* in the Symbiodiniaceae phycosphere.

*Roseibium* sp. strain Sym1 was isolated (6) from a 0.45-μm filtrate slurry of a 3-year, xenic monoculture of Symbiodinium linucheae, an algal symbiont isolated (7) from the anemone *Aiptasia* (*Aiptasia pallida sensu stricto*) (8). Briefly, the algal cells were ruptured with 4.5 mm beads and vortexed at full speed for 90 s. Approximately 1 mL of the slurry was passed through a polyethersulfone (PES) syringe filter (pore size, 0.45 μm; filter diameter, 30 mm) into a clean microcentrifuge tube and subsequently diluted 1:100 in autoclaved filtered seawater. The diluted filtrate (100 μL) was then plated on marine Reasoner’s 2A (R2A) agar. A single colony was picked and subcultured three times to verify that it was a pure isolate. The 16S rRNA gene was amplified using the primer pair 27F/1492R (9), followed by Sanger sequencing (Laragen, Inc., USA), and queried against the NCBI nucleotide database using blastn. The top match was *Labrenzia* sp. strain 12b (identity, 99.49%; GenBank accession number LT629936.1).

*Roseibium* sp. Sym1 was revived from a cryostock and inoculated into 25 mL of marine R2A broth. Once high density was achieved, 25 μL of the seed culture was used to inoculate 25 mL of marine R2A broth in triplicate into cell culture flasks for 72 h at 27°C. Inoculum from one flask was used for paired-end sequencing, and inocula from the other two flasks were used for long-read sequencing. Genomic DNA was extracted using the GenElute bacterial genomic DNA kit (Millipore Sigma, USA). A paired-end library was constructed using the Illumina DNA library prep kit and sequenced on the NextSeq 2000 platform, generating an output of 21,815,230 raw short reads (2 × 151 bp). Oxford Nanopore’s genomic DNA by ligation kit was used for long-read library preparation (no size selection); the library was sequenced on the MinION platform using an R9 flow cell chemistry (R9.4.1), followed by high-accuracy base calling using Guppy v5.0.16, which generated an output of 165,651 raw reads with an *N*_50_ value of 8,039 bp. Library preparation and sequencing were conducted by SeqCenter (USA). Quality control was conducted using fastp v0.12.4 (10) for the short-read sequences and NanoFilt v2.8.0-0 (11) for the long-read sequences. The Unicycler v0.5.0 assembly pipeline (12) was used to generate a hybrid assembly, resulting in five contigs.

The final mean coverage for the genome was estimated to be 55× (using Bowtie v0.7.17 and SAMtools v1.15.1 [13, 14]). Using CheckM v1.2.1 (15), it was determined that the genome is 99.58% complete and has a GC content of 61.2%. The hybrid assembly identified five replicons, consisting of one chromosome and four plasmids (rotated and circularized using Unicycler), totaling 7,257,095 bp. Default parameters were used for all software unless stated otherwise.

### Data availability.

The hybrid assembly is available at NCBI GenBank under accession numbers CP114786, CP114787, CP114788, CP114789, and CP114790. The BioProject accession number is PRJNA884430. The code used to assemble the genome is available at https://github.com/symbiotic-em.
